# Simultaneous Occurrence of Ocular, Disseminated Mucocutaneous, and Multivisceral Involvement of Leishmaniasis

**DOI:** 10.1155/2014/837625

**Published:** 2014-02-18

**Authors:** Cyriac Abby Philips, Chetan Ramesh Kalal, K. N. Chandan Kumar, Chhagan Bihari, Shiv Kumar Sarin

**Affiliations:** ^1^Department of Hepatology, Institute of Liver and Biliary Sciences, D-1, Vasant Kunj, New Delhi 110070, India; ^2^Department of Pathology, Institute of Liver and Biliary Sciences, D-1, Vasant Kunj, New Delhi 110070, India

## Abstract

Leishmaniasis is a tropical infection caused by the protozoan, belonging to the group of *Leishmania* which causes Old World and New World disease. These are typically divided into cutaneous, mucocutaneous, visceral, viscerotropic, and disseminated disease. Cutaneous leishmaniasis in the presence of visceral disease is a rarity. Isolated case reports have documented this occurrence, in the immunocompromised setting, and few otherwise. The concurrent presence of visceral leishmaniasis (bone marrow involvement) with solitary cutaneous and ocular disease and also solitary cutaneous and visceral disease (bone marrow involvement) has been reported before. Here, we present an immunocompetent patient who was diagnosed to have visceral leishmaniasis (liver and bone marrow involvement) along with simultaneous disseminated mucocutaneous and ocular involvement, a combination that has never been reported before.

## 1. Introduction

Leishmaniasis, caused by an obligate intramacrophage protozoan parasite, belonging to the group *Leishmania*, is a vector borne disease affecting human population where the parasite, the animal reservoir, and the vector coexist. Sandflies, belonging to the *Phlebotomus* species, are the vectors responsible for this illness. Leishmaniasis exists in three forms. The less severe form called cutaneous leishmaniasis affects the skin, producing characteristic lesions that can be well managed with treatment. The second form is the more severe visceral leishmaniasis which affects internal organs and can prove to be fatal if not judiciously treated. A third form known as the mucocutaneous leishmaniasis affects skin and mucous membranes of the face, producing ulcerative and disfiguring lesions [1]. Leishmaniasis is commonly seen in the regions of Nepal, India, Bangladesh, and the Middle East. *Leishmania* is a genus of trypanosomatid protozoa and is spread through bite of sandflies of the genus *Phlebotomus* in the Old World and genus *Lutzomyia* in the New World. The cutaneous forms of Leishmaniasis are further characterized into classical cutaneous leishmaniasis, diffuse cutaneous, leishmaniasis recidivans, and post kala-azar dermal leishmaniasis [2]. The other forms include the classical visceral type and viscerotropic type. Concurrent occurrence of these different leishmanial forms in a single host has been reported very rarely in literature [3]. The presence of cutaneous and visceral leishmaniasis has been reported from few countries, mostly in immunocompromised patients. The presence of different forms of leishmaniasis in an immunocompetent host has been reported quite rarely.

## 2. Case Report

A 60-year-old woman, hailing from the eastern state of Uttar Pradesh in India presented to us with six- week history of high grade continuous fever associated with chills, rigors, anorexia, malaise, joint pains, and severe lethargy. The patient also stated that she noticed crops of round, raised lesions that started on her left upper limb, progressing to the right upper limb and eventually involving the whole face, upper back, and the chest and upper abdominal region, closely associated with the febrile episodes. Some of these lesions progressed to increase in size, became more nodular, and developed sunken centers that drained pus-like material, associated with pain and redness. The patient developed jaundice one week preceding her admission to our center which was associated with mild to moderate upper quadrant abdominal pain. There was no history of pruritis, pale-colored stools, or bleeding diathesis. The patient also developed swelling of the legs, which was nonpainful, not associated with facial puffiness, and not in lieu with decrement in urine output. This was associated with painful red eyes and nodular lesions over the eye lids and in the conjunctiva of the eyes associated with decreased vision. She is a teetotaler with no known comorbidities in the past or at the present and has not been consuming over the counter medications and/or complementary and alternative medications with/without steroid combinations or on any other potentially immunosuppressive medications.

**Figure 1:**
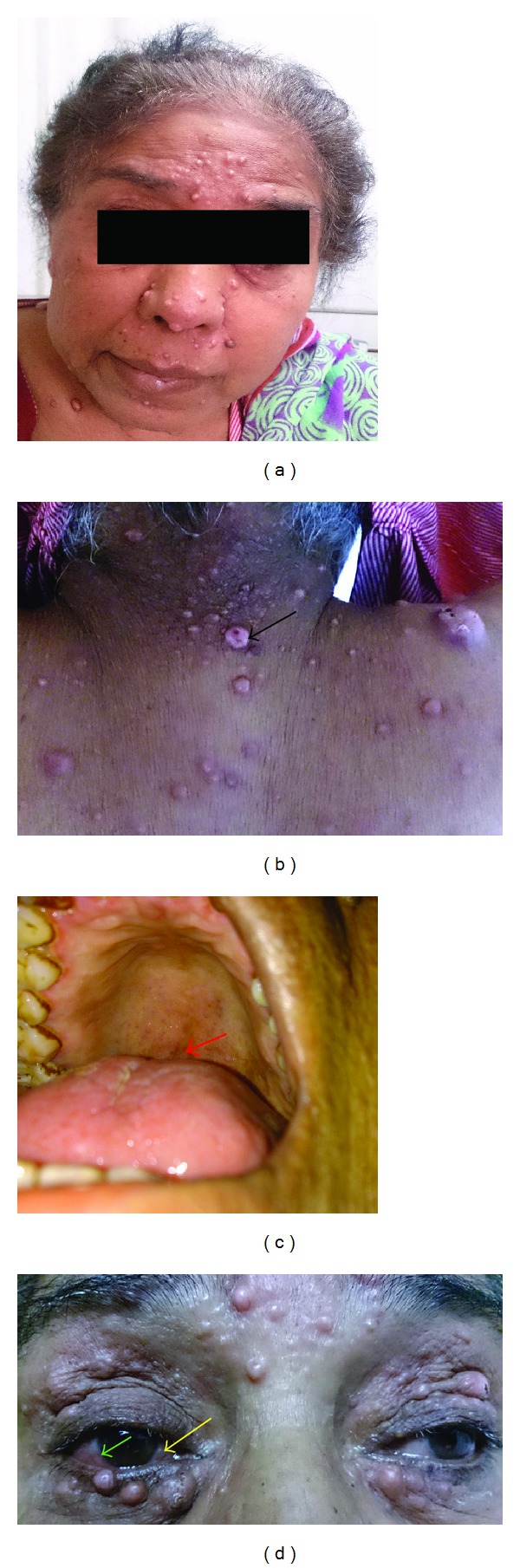
Clockwise, from left: nodular lesions involving the face in a disseminated pattern, mimicking lepromatous leprosy; the boggy pus draining lesions at the back with ulceronodular features (black arrow); eye involvement in the form of nodules on the conjunctiva (yellow arrow) and conjunctival suffusion (green arrow); mucous surface involvement in the form of broken down nodular lesion on the soft palate (red arrow).

On examination, the patient was found to have a temperature of 100.8 F. Her blood pressure was 128/88 mm of Hg in the right brachial region in supine position and the heart rate was 100 per minute. Pallor was evident; there was an icteric tinge without clubbing, lymphadenopathy, or cyanosis. There was bilateral pedal, pitting, nontender, and symmetrical edema. The skin examination revealed the presence of multiple crops of nodular, noduloulcerative, and papular lesions, dispersed throughout the body surface, mostly segregated to the face and upper back. These lesions were shiny and without skin changes and a few of them showed pus drainage from the central sunken areas (Figure 1). The eye examination revealed the presence of nodular lesions over the eyes and conjunctiva associated with conjunctival suffusion. The abdominal examination revealed the presence of hepatosplenomegaly and no free fluid in the abdomen. The rest of the systemic examination was essentially normal.

**Figure 2:**
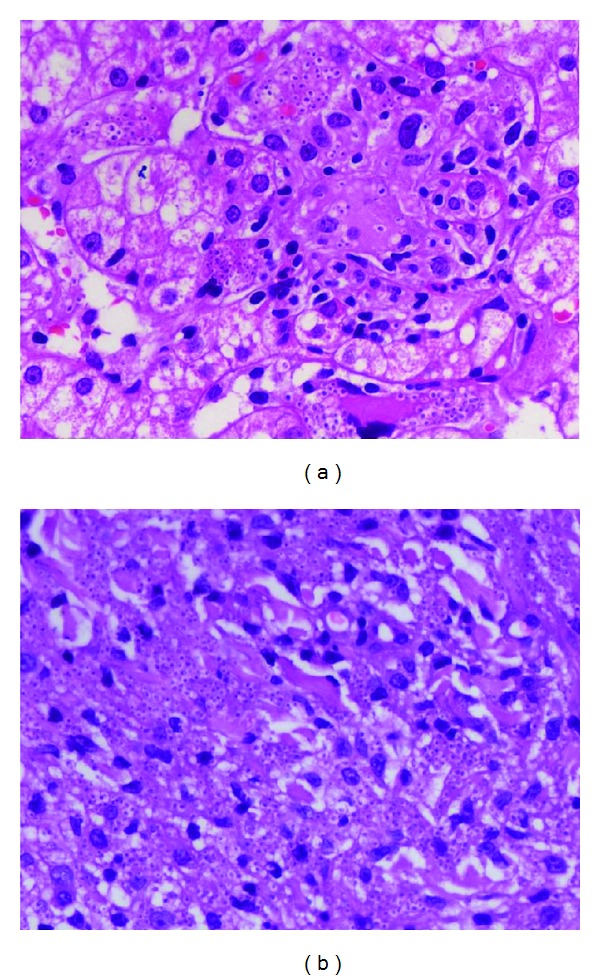
Histopathological examination of the liver (a) and skin lesion (b) showing features of LD bodies in the former and amastigote forms in the latter. H&E stain (400x) and eosin stain (400x), respectively.

**Figure 3:**
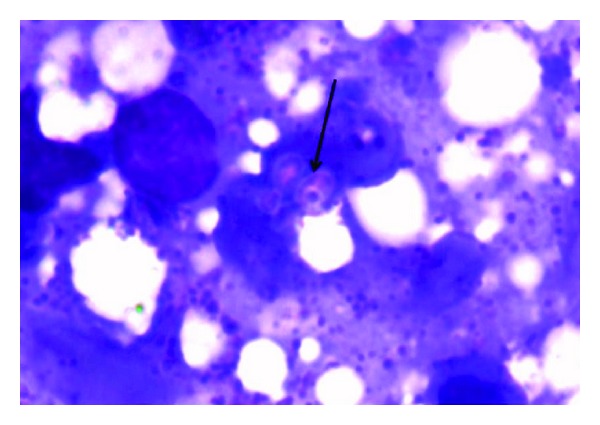
Histopathological examination of the bone marrow showing LD bodies within the histiocytic macrophages (black arrow); eosin stain (1000x).

The blood investigations revealed the presence of hemoglobin of 10.5 g/dL (normal 12 to 15), a total leucocyte count of 5600 per cubic millimeter (normal 4000 to 10000), platelet count of 80000 lakhs per cubic millimeter (1.5 lakhs to 4.5), and an ESR of 80 (normal 0–2) with normocytic normochromic anemia. Her liver function tests revealed a total bilirubin of 3.2 mg/dL (normal 0.3 to 1.2), of which the direct fraction was 1.8 mg/dL (normal 0.2), the aspartate aminotransferase level was 50 IU/L (normal 5–40), alanine aminotransferase was 60 IU/L (7–35), alkaline phosphatase was 221 IU/L (normal 32–92), and gamma glutamyl transpeptidase was 150 IU/L (normal 7 to 64) with albumin value of 1.9 g/dL (normal 3.2 to 4.6) and INR of 1.3. The serum IgG levels were 22.6 g/L (normal 6.39 to 13.49). HIV 1 and 2, HBsAg, and anti-HCV were negative and an abdominal imaging revealed the presence of only hepatosplenomegaly without ascites or intrahepatic biliary radical dilatation and absence of abdominal lymphadenopathy. A fundoscopy performed at the bedside revealed features of uveitis and small retinal hemorrhages. The imaging of the chest was within normal limits. The patient underwent a skin biopsy from the largest lesion at the back; a percutaneous ultrasound guided liver biopsy was also done and a subsequent bone marrow study was also undertaken. The findings of the skin biopsy suggested the presence of thinned out epidermis. The dermis showed loss of adnexal structures and a rich collection of histiocytes filled with amastigote forms of leishmaniasis (Figure 2(a)). The liver biopsy revealed the presence of acinar disarray with hepatocytes showing ballooning degeneration, mild steatosis, and glycogenated nuclei. Several foci of lobular inflammation and prominence of hyperplastic Kupffer cells were noted with formation of few small granulomas. The hyperplastic Kupffer cells and granulomatous collection of macrophages showed presence of intracellular LD bodies (Figure 2(b)) which was also subsequently shown in the bone marrow biopsy (Figure 3). The patient was then finally diagnosed as a case of disseminated mucocutaneous-cutaneous leishmaniasis with concurrent ocular, hepatic, and simultaneous bone marrow involvement. She was started on antileishmanial therapy with liposomal amphotericin B at a dose of 3 mg/kg body weight from days 1 to 10 and oral Miltefosine of 100 mg per day for the next 28 days. The laboratory parameters subsequently normalized and the patient's visual acuity and other symptoms improved substantially during the rest of her hospital stay. She was discharged eventually in a stable condition, being afebrile, and is currently on regular follow up.

## 3. Discussion

Leishmaniasis is a protozoan disease that is categorized into Old World disease (being prevalent in Africa, Middle East, Asia, and the Mediterranean) and New World disease (in areas of Central and South America). The Old World species produce, mostly, disseminated skin disease rather than solitary skin disease. These organisms cause cutaneous, mucocutaneous, visceral, and disseminated diseases. Cutaneous leishmaniasis can be divided into localized, diffuse, recidivans, post kala azar dermal and mucocutaneous types. Localized form resembles lepromatous type of Hansen's disease with ≥10 lesions at multiple body sites. The recidivans type resembles lupus vulgaris with psoriasiform plaques that occur on the face, progressing centrifugally, many months after the resolution of solitary cutaneous disease. Post kala-azar dermal leishmaniasis is seen to complicate a resolved visceral lesihmaniasis and finally, mucocutaneous Leishmaniasis affects both the mucosal surfaces as well as skin areas of the face, forming disfiguring lesions. The visceral leishmaniasis involves the visceral organs, mainly the liver and spleen and bone marrow presenting mostly with fever and severe malaise associated with pancytopenia and hypergammaglobulinemia with pigmentation and xerosis of the skin [4]. Another variety, known as the viscerotropic form, caused by the species *Leishmania tropica* (which mostly produces cutaneous form of the disease) has been described from India and Israel. In this form, the patient does not have the full-fledged form of visceral disease but rather has symptoms of diarrhea, malaise, and abdominal pain with loss of general well-being, with a prolonged and indolent course. The diagnosis of this disease relies mostly on the demonstration of the protozoan in tissue samples like aspirates and biopsy samples. Imaging has very minimal role in confirmation of Leishmaniasis. Montenegro leishmaniasis skin test and cellulose acetate electrophoresis (for typing the protozoan species) have been used previously. This has been replaced by the new serological test, RK39, and PC R methods to detect the species of infecting organism [5, 6]. The simultaneous occurrence of different forms of leishmaniasis in a single host is very rare. There have been isolated case reports of concurrent forms of leishmaniasis occurring in patients. Gontijo et al. reported the presence of cutaneous, visceral, and ocular leishmaniasis in a single host, postrenal transplantation [7]. Atypical forms of leishmaniasis and concurrent forms have been known to occur in immunocompromised patients [8]. Our patient was immunocompetent and she has had no other comorbidities, which makes this presentation even more peculiar. Visceral leishmaniasis, traditionally, is not associated with cutaneous lesions. Ben-Ami et al. had reported the occurrence of adult visceral leishmaniasis concurrently with a single cutaneous lesion. In their patient, they had demonstrated the presence of *Leishmania donovani* complex in skin and bone marrow samples [9]. In our patient, we could demonstrate the presence of amastigote forms of *Leishmania* in the skin and liver and of LD bodies in the bone marrow with concurrent ocular involvement with active fundoscopic changes. This spectrum of multiple organ involvement in leishmaniasis has never been reported before. Even more striking in our patient was that the type of cutaneous lesions seen was of the disseminated type, closely resembling lepromatous leprosy. Hence, this concurrent occurrence of disseminated mucocutaneous leishmaniasis, with ocular, liver, and bone marrow involvement in a fully immunocompetent patient would probably be the first of its kind. Mings et al. had reported the presence of “boggy” indurated swellings in cutaneous leishmaniasis associated with mucosal involvement [10]. This changing type of skin lesions in the course of disease has been noted in our patient also. Our patient had unusual boggy, centrally sunken nodular lesions along with the typical ulcerative and ulceronodular lesions. Schnur et al. had described unusual leishmanial strain from Israel with multifarious characterization [11]. Faiman et al. also recently published a series that shed light on the emergence of a novel cutaneous leishmaniasis pattern in Israel [12]. This brings into light the possibility of changing pattern of disease phenotype and protozoan characteristics and eventual manifestation in the host, for which, further work, both at the host level and at the protozoan level, is warranted.
